# A Rare Complication of a Vaginal Breech Delivery

**DOI:** 10.1155/2011/306124

**Published:** 2011-09-08

**Authors:** H. C. Vergers-Spooren, J. W. de Leeuw

**Affiliations:** ^1^Department of Obstetrics and Gynaecology, Sint Franciscus Gasthuis, Kleiweg 500, 3045 PM Rotterdam, The Netherlands; ^2^Department of Obstetrics and Gynecology, Ikazia Ziekenhuis, 3083 AN Rotterdam, The Netherlands

## Abstract

Rectal lesions without anal sphincter trauma in childbirth are only sporadically described in literature. We describe the case of a 29-year-old primigravida who delivered a child in frank breech presentation. During the second stage of labour a foot presented transanally through a rectal laceration with intact anal sphincters. The laceration was repaired immediately after delivery in theatre. Follow-up visits showed a properly cured laceration and no complaints of incontinence or foul discharge.

## 1. Introduction

Obstetric perineal and anal sphincter injuries have been well described. Perineal tears are divided into four grades according to the extent of the lesion [1]. There is no separate classification for obstetrical rectal tears without injury to the perineum. To our knowledge the prevalence of these injuries has not been reported in the literature. Failure to recognize and repair perineal and rectal lacerations is associated with short and long-term morbidity as hemorrhage, perineal pain, dyspareunia, rectovaginal fistulae, perineal abscess, and incontinence of stool or flatus [2]. With this case report, we would like to make the readers aware that a rectal lesion is a potential but rare complication of vaginal delivery. If it is recognised and treated properly, uneventful recovery is very well possible.

## 2. Case Presentation

A 29-year-old primigravida was admitted to the labour room at 38 5/7 weeks amenorrhea for delivery with frank breech presentation. She was referred to us from primary care by the independent midwife at a gestational age of 35 weeks because of breech presentation. Her medical history was uneventful. External cephalic version was unsuccessful, and after counselling a vaginal delivery was accepted.

**Figure 1:**
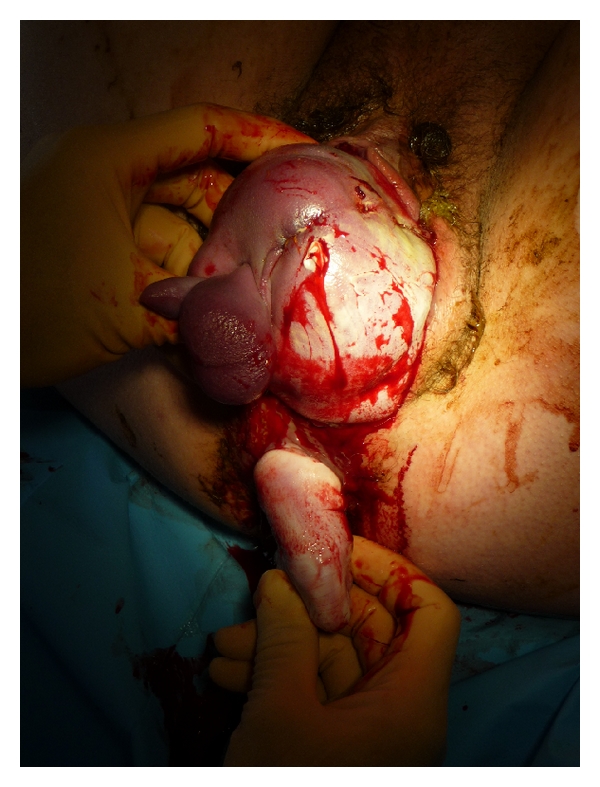
Left leg born to rectovaginal septum and anus.

The first stage of labour progressed normally with support of low-dose oxytocin. Second stage of labour started after full dilatation was reached with the breech at level Hodge 3. A left mediolateral episiotomy was placed when the foetal anus remained visible in the vaginal introitus between contractions. Before the start of the next contraction, the left foot and lower part of the left leg of the baby was born through the rectovaginal septum and the anus. (Figure 1). Immediately we instructed our patient not to push during the next contraction. The left leg was carefully pulled back through the rectovaginal septum by flexion of the leg in the hollow of the knee and careful pushing upwards, of the foot through the anus towards the vagina with the accoucheurs other hand. 

After secondary breech extraction using the Løvset and Mauriceau maneuver, a boy of 2860 grams was born with an Apgar score of 6 after 1 minute and 9 after 5 minutes and umbilical artery pH of 7.06 and BE of −11.6. After short resuscitation with three rescue breaths, he recovered spontaneously and developed well during the first months.

Rectovaginal examination immediately after delivery showed a “button-hole lesion” of the rectovaginal septum of 2 to 3 centimetres length situated one centimetre cranial of the anal canal and just medial of the vaginal part of the episiotomy. Both the internal and external sphincter appeared to be intact. The laceration was repaired under general anaesthesia in theatre. The rectal mucosa and rectovaginal septum were sutured with interrupted Monocryl 4-0 (poliglecaprone 25) sutures. The vaginal mucosa was sutured with continuous Vicryl 2-0 sutures. The episiotomy was then repaired routinely according to our local protocol. Amoxycilline/Clavulanic acid (Augmentin) was given intravenously as prophylaxis. Postoperatively, oral magnesiumoxide was given as stool softener. Both the rectovaginal tear and the episiotomy healed primarily. 

At the follow-up visit after 6 weeks, and after three months, our patient had no complaints of incontinence or foul discharge. Rectovaginal examination, including endo-anal ultrasound, showed a properly cured laceration and episiotomy. Both anal sphincters appeared to be intact.

## 3. Discussion

A review of the literature revealed very few cases reporting an isolated rectal lesion during parturition [3–7]. This may be because of its rarity, but the most possible explanation is underreporting of the problem. A rectal lesion due to perforation of the rectovaginal septum by the foot of the child is, to our knowledge, only twice described. Lesh [4] described a case in 1952 and a second case is described as “hear-say” in the textbook on Operative Obstetrics by Myerscough [8]. Isolated rectal lesions during parturition by other causes were found in seven other cases [3, 5–7]. Three were after vacuum delivery, three during spontaneous delivery, and one prior to the first traction in vacuum delivery. All these cases concerned deliveries with the child in vertex presentation [3, 5–7]. In some cases the colorectal surgeon was involved in the immediate repair, but in none of these cases a diverting colostomy was made. 

It is important to recognize obstetric anal sphincter and rectal injuries. These lesions can be missed if rectal examination is not carried out as a standard procedure prior to suturing. This can have a devastating effect on the physical and emotional well-being of women. Rosenhein et al. [9] give a classification of lesions (not necessarily obstetric in origin) and give a possible protocol for treatment. The lesion in our patient was recognized in the acute period. It was possible to repair it directly with no long-term complications. When repairing these lesions, adequate visualization is essential and general or regional (spinal or epidural) anaesthesia is therefore mandatory. Creating an artificial fourth degree sphincter lesion is not required as long as the exposure is adequate. Incising the anal sphincter creates a risk for possible anal dysfunction, even if the lesion is repaired adequately.

 Several factors may play a role in the aetiology of these lesions, including instrumentation, birth weight of more than 4 kilograms, persistent occipitoposterior presentation, nulliparity, tissue factors, and second stage >1 hour [10]. It is also postulated that rapid descent of the foetal head gives inadequate time for tissues to adjust to the passage of the foetus. The only apparent predisposing factor in our patient was nulliparity. 

Our patient and the presented cases in the literature show that uneventful recovery of an isolated rectal lesion is very well possible, without the necessity of a diverting colostomy or incision of the perineum through the anal canal, if recognised and repaired immediately.
